# HSP70-Mediated NLRP3 Inflammasome Suppression Underlies Reversal of Acute Kidney Injury Following Extracellular Vesicle and Focused Ultrasound Combination Therapy

**DOI:** 10.3390/ijms21114085

**Published:** 2020-06-08

**Authors:** Mujib Ullah, Daniel D. Liu, Sravanthi Rai, Waldo Concepcion, Avnesh S. Thakor

**Affiliations:** 1 Interventional Regenerative Medicine and Imaging Laboratory, Department of Radiology, Stanford University School of Medicine, Palo Alto, CA 94304, USA; ullah@stanford.edu (M.U.); liudan@stanford.edu (D.D.L.); drrai@stanford.edu (S.R.); 2 Department of Surgery, Stanford University School of Medicine, Palo Alto, CA 94304, USA; wconcepcion@stanfordmed.org

**Keywords:** inflammasome, heat shock protein, extracellular vesicles, focused ultrasound, mesenchymal stromal cells, acute kidney injury, regenerative medicine

## Abstract

Acute kidney injury (AKI) is the abrupt loss of renal function, for which only supportive therapies exist. Mesenchymal stromal cell (MSC)-derived extracellular vesicles (EVs) have been shown to be therapeutically effective in treating AKI by spurring endogenous cell proliferation and survival while suppressing inflammation. Pre-treating kidneys with pulsed focused ultrasound (pFUS) has also been shown to enhance MSC therapy for AKI, but its role in MSC-derived EV therapy remains unexplored. Using a mouse model of cisplatin-induced AKI, we show that combination therapy with pFUS and EVs restores physiological and molecular markers of kidney function, more so than either alone. Both pFUS and EVs downregulate heat shock protein 70 (HSP70), the NLRP3 inflammasome, and its downstream pro-inflammatory cytokines IL-1β and IL-18, all of which are highly upregulated in AKI. In vitro knockdown studies suggest that HSP70 is a positive regulator of the NLRP3 inflammasome. Our study therefore demonstrates the ability of pFUS to enhance EV therapy for AKI and provides further mechanistic understanding of their anti-inflammatory and regenerative effects.

## 1. Introduction

Acute kidney injury (AKI) is the sudden loss of renal function, usually due to ischemia, nephrotoxic agents, or urinary tract obstructions [1]. Although AKI is a relatively common condition, especially in hospitalized and chronically ill patients, treatments remain largely supportive, despite mortality associated with this condition being as high as 20% [2]. Hence, there is growing interest in developing regenerative therapies for AKI that can repair renal injury as well as prevent its progression to chronic kidney disease.

AKI is associated with both systemic and intrarenal inflammation, which are believed to be key components underlying its pathophysiology [3]. Although inflammation in the acute phase can facilitate tissue repair following injury, disruption of this process can lead to persistent inflammation, causing tissue damage and fibrosis [4]. Many molecular mediators of inflammation have been identified in AKI [5], which include the NLRP3 inflammasome [6], toll-like receptors (TLRs) [7], and various secreted cytokines that promote neutrophil- and monocyte-mediated inflammatory responses [5,8]. Indeed, blockade of innate immune receptors seems to confer protection against AKI in several preclinical studies [9,10,11,12].

Another therapeutic strategy for immune modulation lies in mesenchymal stromal cell (MSC)-based therapies [13]. MSCs are multipotent cells that have been investigated as a cell therapy for regenerative medicine applications, including AKI [14,15]. Their therapeutic effect arises from their ability to home to damaged tissue and secrete extracellular vesicles (EVs) and other factors that act in a paracrine manner to exert proliferative, pro-survival, and anti-inflammatory effects [16,17,18,19]. More recent studies have begun exploring purified MSC-derived EVs as a cell-free alternative to MSC therapy [20,21,22]; the advantages of using EVs compared to MSCs include their higher safety profile, ability to cross barriers with minimal sequestration in the pulmonary microvasculature following intravenous infusion, lower immunogenicity, and avoidance of complications related to stem cell-induced tumor formation [23,24,25,26,27,28].

While EVs can achieve a therapeutic effect comparable to their parent MSCs in the context of AKI, there remains great interest in optimizing their efficacy. Pulsed focused ultrasound (pFUS), where target organs are selectively treated with focused sound waves, has recently emerged as a method to improve MSC-based therapies [29]. Pre-treatment of target organs with pFUS has been shown to locally upregulate cytokines and trophic factors, improve MSC homing, and subsequently their therapeutic efficacy [30,31,32]. However, the full range of mechanisms underlying pFUS has yet to be elucidated [29,33], and its effect on EV therapy is particularly lacking. Here, we assess the effect of combination therapy with pFUS and MSC-derived EVs in a mouse model of cisplatin-induced AKI, evaluating in particular their ability to suppress AKI-related inflammation.

## 2. Results

### 2.1. Reversal of AKI Using EVs and pFUS

**Figure 1:**
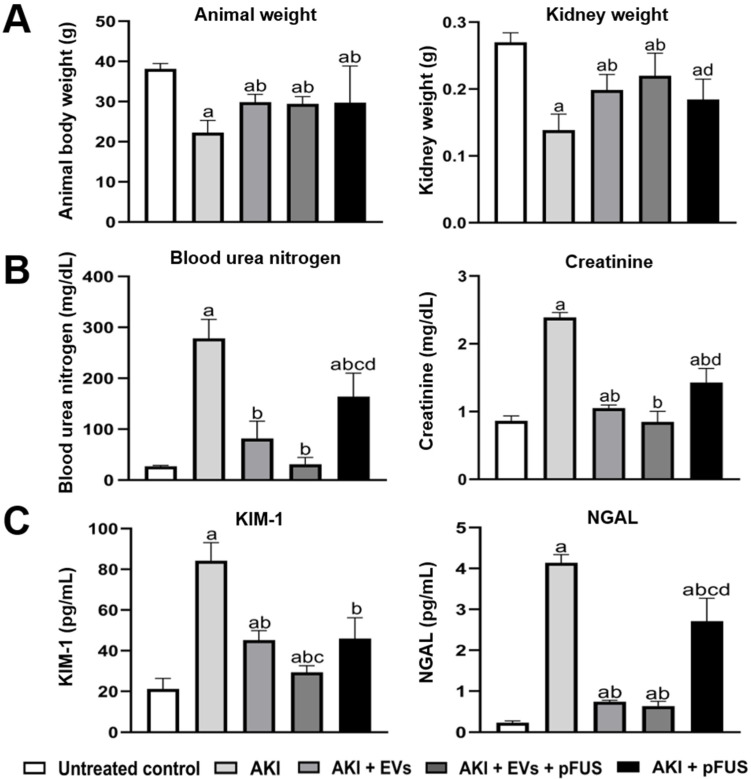
Physiological and biochemical measures of kidney function. (**A**) Animal body weight and kidney weight. (**B**) ELISA of blood urea nitrogen (BUN) and serum creatinine. (**C**) ELISA of molecular kidney injury markers KIM-1 and NGAL. Measurements were taken 9 days following cisplatin treatment. Each group has *n* = 5 mice. Significant difference ^a^ *p* < 0.05 relative to untreated control; ^b^ *p* < 0.05 relative to AKI; ^c^ *p* < 0.05 relative to AKI + EVs; ^d^ *p* < 0.05 relative to AKI + EVs + pFUS.

Following the induction of AKI using cisplatin at day zero, mice were either administered pFUS at day 2, EVs at day 3, or a combination of both. Compared to untreated controls, mice with AKI showed a significant decrease in the animal body weight (38.14 ± 1.35 vs. 22.29 ± 3.04 g, *p* < 0.05) and kidney weight (0.27 ± 0.01 vs. 0.14 ± 0.02 g, *p* < 0.05) (Figure 1A), significant increases in blood urea nitrogen (BUN) (27.27 ± 1.52 vs. 278.48 ± 37.29 mg/dL, *p* < 0.05) and serum creatinine (SCr) (0.86 ± 0.07 mg/dL vs. 2.39 ± 0.07, *p* < 0.05) (Figure 1B), and significant increases in serum concentration of molecular injury markers KIM-1 (21.17 ± 5.17 vs. 84.19 ± 8.96 pg/mL, *p* < 0.05) and NGAL (0.23 ± 0.04 vs. 4.14 ± 0.20 pg/mL, *p* < 0.05) (Figure 1C).

Compared to mice in the AKI group, those treated with pFUS alone demonstrated a significant increase in body weight (22.29 ± 3.04 vs. 29.71 ± 9.15 g, *p* < 0.05) and non-significant increase in kidney weight (0.14 ± 0.02 vs. 0.18 ± 0.03 g, *p* > 0.05) (Figure 1A), a significant decrease in BUN (278.48 ± 37.29 vs. 164.33 ± 45.74 mg/dL, *p* < 0.05) and SCr (2.39 ± 0.07 vs. 1.43 ± 0.21 mg/dL, *p* < 0.05) (Figure 1B), and a significant decrease in serum KIM-1 (84.19 ± 8.96 vs. 45.95 ± 10.28 pg/mL, *p* < 0.05) and NGAL (4.14 ± 0.20 vs. 2.71 ± 0.56 pg/mL, *p* < 0.05) (Figure 1C).

Compared to mice in the AKI group, those treated with EVs alone also demonstrated a significant increase in body weight (22.29 ± 3.04 vs. 29.85 ± 1.95 g, *p* < 0.05) and kidney weight (0.14 ± 0.02 vs. 0.20 ± 0.02 g, *p* < 0.05) (Figure 1A), a significant decrease in BUN (278.48 ± 37.29 vs. 81.89 ± 34.11 mg/dL, *p* < 0.05) and SCr (2.39 ± 0.07 vs. 1.05 ± 0.05 mg/dL, *p* < 0.05) (Figure 1B), and a significant decrease in serum KIM-1 (84.19 ± 8.96 pg/mL vs. 45.18 ± 4.71, *p* < 0.05) and NGAL (4.14 ± 0.20 pg/mL vs. 0.64 ± 0.11, *p* < 0.05) (Figure 1C). Overall, the effect of EVs alone was more pronounced on than that of pFUS alone.

Notably, combined treatment with EVs and pFUS often had a greater effect than either alone. Compared to those treated with EVs alone, mice treated with pFUS + EVs had higher kidney weight (0.20 ± 0.02 vs. 0.22 ± 0.03 g, *p* > 0.05) (Figure 1A), lower BUN (81.89 ± 34.11 vs. 31.00 ± 13.61 mg/dL, *p* > 0.05) and SCr (1.05 ± 0.05 vs. 0.85 ± 0.15 mg/dL, *p* > 0.05) (Figure 1B), and lower serum levels of KIM-1 (45.18 ± 4.71 vs. 29.36 ± 3.29 pg/mL, *p* < 0.05) and NGAL (0.75 ± 0.03 vs. 0.64 ± 0.11, *p* > 0.05) (Figure 1C). Though these differences often did not reach statistical significance, there was a consistent trend for all measured physiological and molecular markers.

### 2.2. HSP70-Mediated Regulation of the NLRP3 Inflammasome

**Figure 2:**
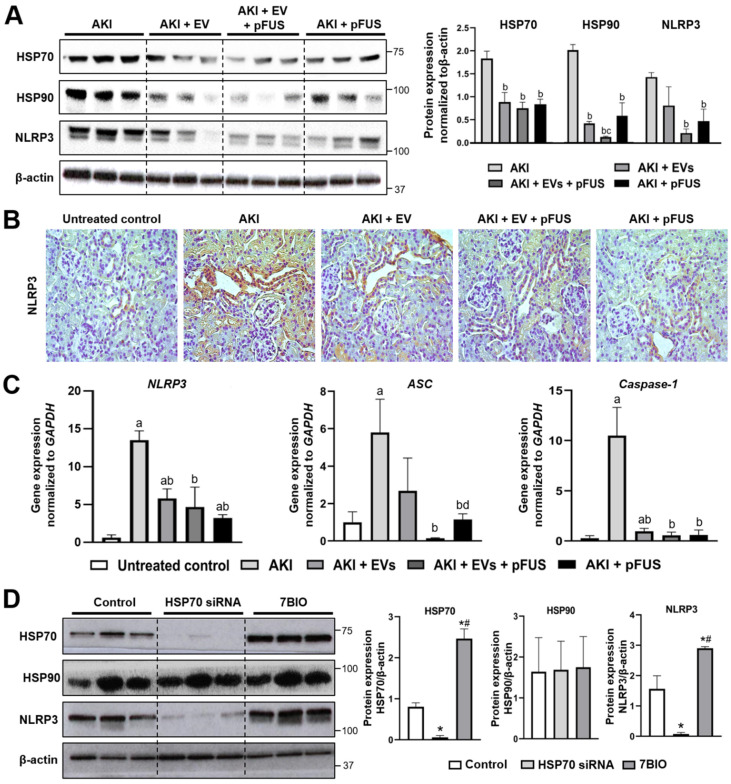
HSP70 regulation of the NLRP3 inflammasome. (**A**) Western blot and quantification showing the expression of HSP70, HSP90, NLRP3, and β-actin in kidney tissue. (**B**) Immunohistochemical staining for NLRP3 in kidney tissue. (**C**) qRT-PCR measurement of inflammasome components *NLRP3*, *ASC*, and *Caspase-1* expression in kidney tissue, normalized to *GAPDH*. (**D**) Western blot and protein quantification showing expression of HSP70, HSP90, NLRP3, and β-actin in human embryonic epithelial (HEK) cells treated with DMSO (control), an HSP70-siRNA, or 7BIO. Each group has *n* = 3 mice. Significant difference ^a^ *p* < 0.05 relative to untreated control; ^b^ *p* < 0.05 relative to AKI; ^c^ *p* < 0.05 relative to AKI + EVs; ^d^ *p* < 0.05 relative to AKI + EVs + pFUS; * *p* < 0.05 relative to DMSO control; ^#^ *p* < 0.05 relative to HSP70-siRNA.

Through Western blot analysis, we found that the heat shock proteins HSP70 and HSP90 are strongly downregulated in the kidneys by both EVs alone (HSP70: 0.89 ± 0.21 vs. 1.23 ± 0.16 normalized expression, *p* < 0.05; HSP90: 0.41 ± 0.04 vs. 1.99 ± 0.12 normalized expression, *p* < 0.05) and by pFUS alone (HSP70: 0.84 ± 0.11 vs. 1.23 ± 0.16 normalized expression, *p* < 0.05; HSP90: 0.58 ± 0.28 vs. 1.99 ± 0.12 normalized expression, *p* < 0.05) compared to the AKI group (Figure 2A). The combined pFUS + EVs treatment downregulated these proteins more so than EVs alone (HSP70: 0.75 ± 0.13 vs. 0.89 ± 0.21 normalized expression, *p* > 0.05; HSP90: 0.12 ± 0.01 vs. 0.41 ± 0.40 normalized expression, *p* < 0.05).

As HSPs are known to regulate inflammation [34], we assessed whether there was any correlation between the expression of HSP70/90 and inflammasome proteins. Compared to the AKI group, NLRP3 was suppressed in the kidneys by both EVs alone (1.42 ± 0.10 vs. 0.81 ± 0.41 normalized expression, *p* > 0.05) and pFUS alone (1.42 ± 0.10 vs. 0.47 ± 0.26 normalized expression, *p* < 0.05) (Figure 2A). The combined pFUS + EV treatment, however, resulted in the most potent suppression of NLRP3 compared the AKI group (1.42 ± 0.10 vs. 0.21 ± 0.09 normalized expression, *p* < 0.05). IHC staining for NLRP3 on kidney tissue recapitulated these findings, with the strongest NLRP3 staining in the AKI condition and markedly reduced staining following treatment with EVs, pFUS, or both (Figure 2B). Similar trends were observed using qRT-PCR analysis of *NLRP3* in kidney lysate, as well as two other inflammasome components, apoptosis-associated speck-like protein containing a CARD (ASC) and Caspase-1 (Figure 2C). Compared to untreated controls, those in the AKI group had significant elevations of *ASC* and *Caspase-1* in the kidney (*ASC*: 1.00 ± 0.56 vs. 5.80 ± 1.78 normalized expression, *p* < 0.05; *Caspase-1*: 0.29 ± 0.24 vs. 10.49 ± 2.82 normalized expression, *p* < 0.05). Compared to the AKI group, expression of these genes was suppressed following treatment with either EVs alone (*ASC*: 5.80 ± 1.78 vs. 2.69 ±1.74 normalized expression, *p* > 0.05; *Caspase-1*: 10.49 ± 2.82 vs. 0.97 ± 0.29 normalized expression, *p* < 0.05) or pFUS alone (*ASC*: 5.80 ± 1.78 vs. 1.15 ± 0.30 normalized expression, *p* < 0.05; *Caspase-1*: 10.49 ± 2.82 vs. 0.60 ± 0.49 normalized expression, *p* < 0.05). Combined treatment with pFUS + EVs resulted in an even greater suppression of these genes compared to EVs alone (*ASC*: 0.14 ± 0.03 vs. 2.69 ± 1.74 normalized expression, *p* > 0.05; *Caspase-1*: 0.57 ± 0.31 vs. 0.96 ± 0.29 normalized expression, *p* > 0.05), though the differences did not reach statistical significance.

We next sought to validate the mechanistic link between HSPs and the NLRP3 inflammasome. HSP90 has already been documented to positively regulate the NLRP3 inflammasome [35,36,37], but HSP70 has a less straightforward role [38,39,40], leading us to further investigate the latter. We treated human embryonic kidney (HEK) cells with either an HSP70 siRNA or with 7BIO (a non-apoptotic cell death inducer). Knockdown of HSP70 suppressed expression of the inflammasome protein NLRP3, while administration of 7BIO upregulated both HSP70 and NLRP3 (Figure 2D), suggesting that HSP70 is a positive regulator of the NLRP3 inflammasome. Neither condition affected HSP90 expression.

### 2.3. Suppression of Inflammation Following EV and pFUS Therapy

**Figure 3:**
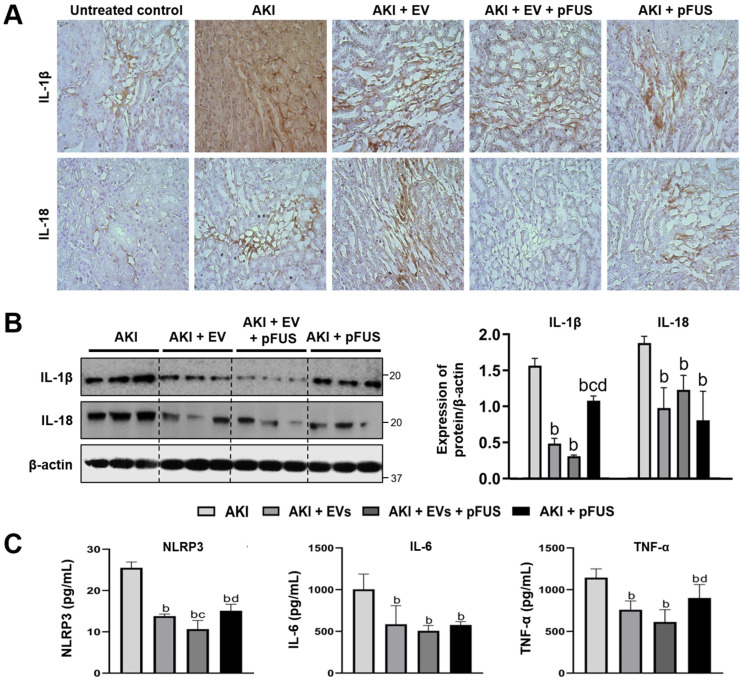
Inflammatory cytokines. (**A**) Immunohistochemical staining for IL-1β and IL-18 on kidney sections. (**B**) Western blot and quantification of IL-1β and IL-18, *n* = 3 for each group. (**C**) ELISA quantification of serum NLRP3, IL-6 and TNF-α, *n* = 5 for each group. Significant difference ^a^ *p* < 0.05 relative to untreated control; ^b^ *p* < 0.05 relative to AKI; ^c^ *p* < 0.05 relative to AKI + EVs; ^d^ *p* < 0.05 relative to AKI + EVs + pFUS.

Given that pFUS and EVs suppress the NLRP3 inflammasome, we next assessed the extent of systemic and intrarenal inflammation following treatment. Using IHC, we found that IL-1β and IL-18, two pro-inflammatory cytokines activated by the NLRP3 inflammasome, were significantly increased in the AKI condition, while treatment with EVs, pFUS, or both reduced their expression (Figure 3A). These results were confirmed by Western blot: compared to the AKI group, IL-1β and IL-18 were suppressed by both EVs alone (IL-1β: 1.57 ± 0.10 vs. 0.48 ± 0.07 relative expression, *p* < 0.05; IL-18: 1.88 ± 0.10 vs. 0.98 ± 0.28 relative expression, *p* < 0.05) and pFUS alone (IL-1β: 1.57 ± 0.10 vs. 1.08 ± 0.07 relative expression, *p* < 0.05; IL-18: 1.88 ± 0.10 vs. 0.81 ± 0.40 relative expression, *p* < 0.05) (Figure 3B). The combined pFUS + EVs treatment resulted in more potent suppression of IL-1β than either EVs alone (0.48 ± 0.07 vs. 0.31 ± 0.01 relative expression, *p* > 0.05) or pFUS alone (1.08 ± 0.07 vs. 0.31 ± 0.01 relative expression, *p* < 0.05). Similar trends were observed using serum ELISA of NLRP3, IL-6, and TNF-α, with significant decreases following treatment with either EVs alone or pFUS alone, and the most potent decreases following combination therapy with pFUS + EVs (Figure 3C).

## 3. Discussion

**Figure 4:**
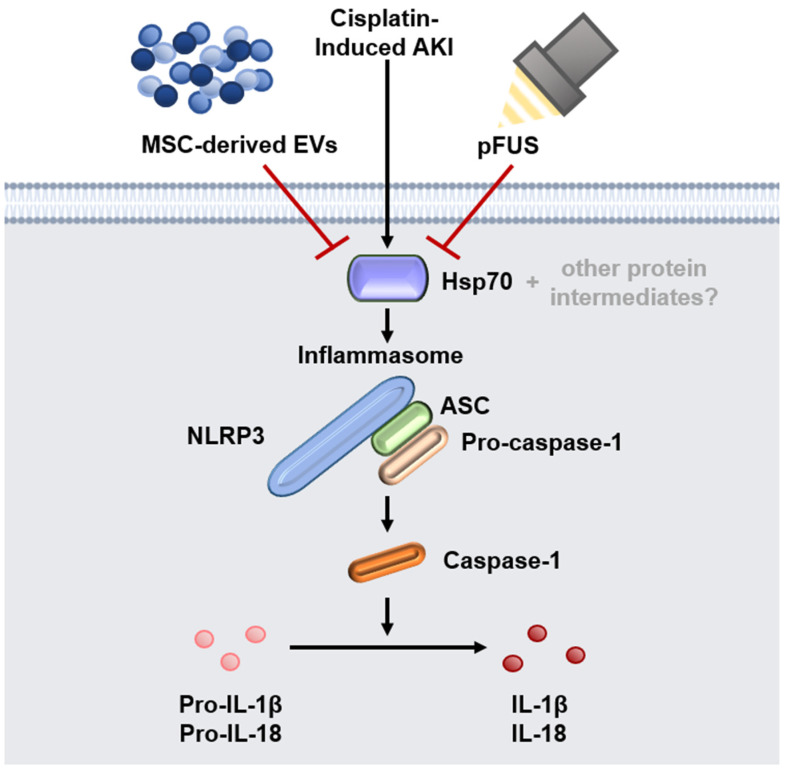
Proposed mechanism of NLRP3 inflammasome suppression. Schematic showing the proposed mechanism by which pFUS and EVs suppress HSP70, the latter of which acts as a positive regulator of the NLRP3 inflammasome.

We have shown that the pretreatment of kidneys suffering from AKI with pFUS enhances the therapeutic effect of MSC-derived EVs. This synergistic effect is at least in part due to downregulation of HSP70, which in turn reduces the formation of the NLRP3 inflammasome, resulting in the attenuation of the pro-inflammatory environment characteristic of AKI (Figure 4).

pFUS is a non-invasive procedure with an excellent safety profile that can be precisely targeted to deep body tissues, and is already FDA-approved for several clinical applications [41]. pFUS has previously been shown to enhance MSC therapy for AKI. However, the mechanism by which this occurs has yet to be fully understood, likely due to differences in ultrasound parameters used between various groups [29]. Some studies have reported that pFUS upregulates local cytokines which serve as a homing signal for MSCs, thereby increasing their accumulation in sonicated tissue and increasing their therapeutic effect [31]. On the other hand, we have previously found that pFUS may have an independent therapeutic effect in AKI, and can enhance MSC therapy independent of increased homing [33]. Consistent with our previous study, we have found here that pFUS is independently able to attenuate NLRP3-mediated inflammation, with subsequent improvements in physiological kidney function. Additionally, we demonstrate that pFUS acts synergistically with EV therapy to reverse AKI.

The NLRP3 inflammasome is an intracellular protein complex consisting of NLRP3, ASC, and pro-caspase-1, which upon activation releases active caspase-1 that proceeds to convert pro-inflammatory cytokines IL-1β and IL-18 into their mature form [42]. The inflammasome has been shown to be upregulated in both mouse models of AKI and human renal biopsies from different pathologies [6]. NLRP3 also has inflammasome-independent effects in tubular epithelial cells [9], including participating in SMAD2 and SMAD3 phosphorylation in response to TGFβ signaling, triggering renal fibrosis [43]. Though there have been previous reports on the suppression of the NLRP3 inflammasome by MSCs [44,45,46] and MSC-derived EVs [47,48], our study is the first to show that pFUS has both an independent and synergistic role in its regulation.

Heat shock proteins (HSPs) are molecular chaperones known to broadly regulate inflammation, including the formation of the NLRP3 inflammasome [34]. However, the exact direction of their regulation appears to be context dependent. HSP90 has been shown to be a positive regulator of the NLRP3 inflammasome in various studies [35,36,37]. HSP70 has also been shown to be a positive regulator of airway inflammation, with HSP70 knockout mice showing significant reductions in airway inflammation compared to wild type mice following intratracheal antigen challenge [39]. Extracellular HSP70 has been shown to act as a cytokine, binding to monocytes through CD14 and activating NF-κB signaling to increase the production of IL-1β, IL-6, and TNF-α [38]. On the contrary, intracellular HSP70 has also been shown to inhibit NLRP3 inflammasome activation in a mouse model of peritonitis [40], where HSP70 deficiency caused worsened NLRP3-dependent peritonitis and enhanced caspase-1 activation and IL-1β production by macrophages, while genetic or heat shock-induced HSP70 overexpression had the opposite effect. The highly context-dependent effects of HSP70 on NLRP3 inflammasome regulation highlights the need for careful studies investigating their molecular links and the involved cell types, details that become crucial should HSP inhibitors be considered for preclinical investigation [49].

Our study has found that in cisplatin-induced AKI, HSP70, HSP90, and NLRP3 are all highly upregulated, and can be suppressed with pFUS and EV therapy. We found that HSP70 knockdown in vitro leads to significant suppression of NLRP3 expression, suggesting HSP70 to be a positive regulator of the NLRP3 inflammasome. We thus propose a mechanism by which pFUS and EVs, likely through intermediate effectors, converge to suppress HSP70, which reduces NLRP3 inflammasome formation and subsequent release of proinflammatory cytokines (Figure 4). Alternative mechanisms must also be considered, including the possibility of another protein targeting both HSP70 and NLRP3. It would be necessary to repeat our experiments in an HSP70 knockout mouse before we can conclude whether the observed therapeutic effect is in fact dependent on HSP70.

In summary, our study demonstrates that pFUS has both independent and synergistic therapeutic effects when used in combination with MSC-derived EVs to treat cisplatin-induced AKI. Both pFUS and EV converge to suppress HSP70/90, which leads to decreased expression of the NLRP3 inflammasome and downstream pro-inflammatory cytokines, ultimately improving kidney function. The growing worldwide prevalence and morbidity of AKI prompts the development of regenerative therapies to restore kidney function and avoid progression to chronic kidney disease. Though EVs have seen substantial preclinical success for AKI, improving their therapeutic efficacy may be necessary for clinical translation. The safety profile and non-invasive nature of pFUS make it an attractive tool, though much remains to be understood about its physiological and molecular effects. Careful characterization of these mechanisms will serve to further its development and optimization as a clinical tool.

## 4. Methods

### 4.1. Animal Experiments

All experimental procedures were performed in accordance with guidelines from the Administrative Panel on Laboratory Animal Care (APLAC) of Stanford University. 40 female CD1 mice were purchased from Charles River Laboratories (Wilmington, MA, USA). Animals were 6 weeks old with body weight in the range of 25–30 g, and were housed for 1 week with 12 h light-dark cycles prior to the start of experiments.

CD1 mice were randomly divided into five groups with 8 animals in each group. Group 1 consisted of untreated control animals. Mice in groups 2–5 received a single intraperitoneal injection of cisplatin (10 mg/kg) on day 0 to induce AKI. Group 2: AKI control, which received no treatment; Group 3: AKI + EVs, which received EVs treatment at a dose of 200 µg/100 g body weight on day 3; Group 4: AKI + EVs + pFUS, which received pFUS on day 2 followed by EVs on day 3; Group 5: AKI + pFUS alone, which received pFUS on day 2. Mice were sacrificed at day 9 after cisplatin injection, at which point the blood and kidney samples were collected. Serum was obtained by centrifugation at 4 °C at 300 × g for 10 min and stored at −20 °C for further analysis. For histological analysis one kidney was immersed in 10% neutral buffered formalin, while the other kidney was frozen in liquid nitrogen for molecular analysis.

### 4.2. Extracellular Vesicle Isolation and Purification

Extracellular vesicles (EVs) were isolated from bone marrow-derived mesenchymal stromal cells (BM-MSCs) pooled from three human donors purchased from ATCC. BM-MSCs were cultured in modified Eagle’s medium (α-MEM) supplemented with 20% fetal bovine serum (FBS) and 100 U/mL penicillin and streptomycin (Thermo Fisher Scientific, Fremont, CA, USA), and incubated at 37 °C with 5% CO_2_. Cells were maintained until passage 3, at which point cells were cultured for 5 more days until they reached 80–90% confluence. Cells were then incubated in fresh serum-free Dulbecco’s modified Eagle medium (DMEM) overnight. The resulting conditioned media was centrifuged at 5000× *g* for 10 min at 25 °C. The supernatant from the previous step was then ultracentrifuged at 17,000× *g* for 20 min. The second supernatant was used to isolate EVs using an anion exchange resin (Q Sepharose Fast Flow, GE Healthcare, Chicago, IL, USA). The resin prepared in three steps: (1) balancing with 50 mM NaCl in 50 mM phosphate buffer, (2) washing with 100 mM NaCl in 50 mM phosphate buffer, and (3) rinsing with 500 mM NaCl in 50 mM phosphate buffer. The supernatant was then applied to the resin. EV fractions were collected, filter sterilized, and stored at 4 °C. EVs were characterized by expression of surface markers (CD9, CD81, TSG101) (Figure S1). EV size was characterized by nanoparticle tracking analysis (size range 20–180 nm, mean 118 nm, standard deviation 27 nm), as well as transmission electron microscopy (TEM), as previous reported [22].

### 4.3. Pulsed Focused Ultrasound

Pulsed focused ultrasound (pFUS) was conducted using a setup of co-aligned transducers with image guidance. pFUS was administered using a 1.1 MHz central frequency custom high-intensity focused ultrasound (HIFU) therapy transducer with 49 mm central opening (H-102NRE, Sonic Concepts, Bothell, WA, USA), with an imaging transducer (Siemens Acuson S2000 14L5 sp, Siemens Corporation, WA, USA) positioned at the central opening of the HIFU transducer. The HIFU transducer was calibrated in a water tank filled with degassed and deionized water as previously described [33]. A custom-made 3D-printed holder was used to align and fix both transducers in place, with the focal spot of the HIFU transducer secured at 55 mm axial and 0 mm lateral to the central point of the imaging transducer. Alignments of the HIFU and imaging beams were checked several times in a water tank containing a hydrophone and oscilloscope. All calibrations resulted in a beam misalignment of less than 200 μm. Mice were anesthetized and submerged vertically, with their heads kept above the water surface. The 3D-printer holder holding both HIFU and imaging transducers was then connected to a translation stage and placed in the water at about 50 mm axial distance from the mice. The imaging transducer was used to identify the mouse’s kidney, and the kidney was placed at the focal spot of the HIFU transducer 55 mm axially and 0 mm laterally from the central point of the imaging transducer. To treat the whole kidney, 8 non-overlapping adjacent regions through the kidney were targeted for 30 s per region. The time to treat one kidney with these parameters was approximately 4 min. In order to deliver pFUS therapy to the animal, the HIFU transducer was used with the following parameters: 5% duty cycle (DC), 5 Hz pulse repetition frequency (PRF), 2.9 MPa peak negative pressure (PNP), and 272 W/cm^2^ spatial average pulse average intensity (I_SAPA_). After pFUS treatment, each mouse was removed from the water bath, dried, and placed in a recovery cage.

### 4.4. Analysis of Kidney Function

ELISA kits were used to measure blood urea nitrogen (BUN) and serum creatinine (SCr) (Santa Cruz Biotechnology, Dallas, TX, USA), serum neutrophil gelatinase-associated lipocalin (NGAL) (R&D Systems, Minneapolis, MN, USA), and kidney injury molecule-1 (KIM-1), NLRP3, IL-6, and TNF-α (Cell Signaling Technology, Danvers, MA, USA). All samples were analyzed according to the manufacturer’s instructions.

### 4.5. Histology, Immunohistochemistry, and Immunofluorescence

Animals were perfused with 4% (vol/vol) paraformaldehyde in PBS, and whole kidney tissues were fixed in formalin for 24 h. Kidney tissues were then sectioned into 6 μm slices for hematoxylin–eosin and trichrome staining. For immunohistochemical staining, first the paraffin embedded sections were deparaffinized, hydrated and antigen-retrieved. Donkey serum was used to block the slides followed by incubation of slides at 4 °C overnight with primary antibodies against NLRP3, IL-1β and Il-18. Later the slides were incubated with secondary antibodies for 1 h and then incubated with 3,3-diaminobenzidine (DAB) (Vector Laboratories, Burlingame, CA, USA) and slides were viewed with a Nikon Eclipse 80i microscope prepared with a digital camera (Nikon, Melville, NY, USA).

### 4.6. NLRP3 Knockdown by siRNA and Overexpression by 7BIO

HEK cells were purchased from the American Type Culture Collection (ATCC, Manassas, VA, USA) and were grown until passage 3 in Dulbecco’s Modified Eagle’s Medium (DMEM) supplemented with 10% fetal bovine serum (FBS), 150 U/mL penicillin, and 150 mg/mL streptomycin. Passage 3 cells were transfected with NLRP3-specific siRNA (final concentration 25 nM; Thermo Fisher Scientific, Fremont, CA, USA) using lipofectamine RNAiMAX transfection reagent (Thermo Fisher Scientific, Fremont, CA, USA) according to the manufacturer’s instructions. Similarly, passage 3 HEK cells were incubated with 5 µM of 7-bromoindirubin-3′-oxime (7BIO) (Bioscience Visions, San Diego, CA, USA) for 24 h, to activate the NLRP3 inflammasome. Protein was isolated from above cells and quantified using Western blot as described below.

### 4.7. Western Blot Analysis

Thin sections of kidney tissue were sonicated and homogenized. The lysate was then placed in 1 × SDS sample buffer in association with radioimmunoprecipitation buffer (RIPA) solution comprising 1% NP40, 0.1% SDS, 100 mg/mL PMSF, 1% protease inhibitor cocktail, and 1% phosphatase I and II inhibitor cocktail (Sigma Aldrich, St Louis, MO, USA) on ice. The supernatant was collected by centrifugation at 13,000× *g* at 4 °C for 30 min. Protein concentration was measured using a bicinchoninic acid protein assay. An equal amount of protein was then loaded into 10% or 15% SDS-PAGE and transferred onto polyvinylidene difluoride membranes. The primary antibodies were as follows: HSP70 (sc-32239, Santa Cruz Biotechnology, Dallas, TX, USA, 1:400 dilution), HSP90 (sc-101494, Santa Cruz Biotechnology, Dallas, TX, USA, 1:400 dilution), NLRP3 (sc06-23, Invitrogen, Waltham, MA, USA, 1:400 dilution), IL-1β (M421B, Thermo Fisher Scientific, Fremont, CA, USA, 1:200 dilution), IL-18 (PA5-79481, Thermo Fisher Scientific, Fremont, CA, USA, 1:200 dilution), anti-β-actin (sc-1616, Santa Cruz Biotechnology, Dallas, TX, USA, 1:200 dilution). Quantification of the Western blot was done by measuring the intensity of the signals using National Institutes of Health Image software package and Bio-Rad image software (Bio-Rad, Hercules, CA, USA).

### 4.8. Quantitative Polymerase Chain Reaction

Extraction of RNA from homogenized kidney tissues was performed using Triazole Reagent (Sigma Aldrich, St Louis, MO, USA) and digested using DNase 1. Reverse transcription was done using reverse transcriptase kit per manufacturer’s instructions (Applied Biosystems, Fremont, CA, USA). cDNA quality was measured by calculating the ratio of absorbance at 260 and 280 nm using an Agilent 2100 Bioanalyzer (Agilent, Santa Clara, CA, USA). Amplification of cDNA was performed using an iCycler Thermal Cycler (Bio-Rad, Hercules, CA, USA) with SYBR Green (Applied Biosystems, Fremont, CA, USA) and specific primers for *NLRP3* (Mm00840904-m1), *ASC* (Mm00445747-g1) and *Caspase-1* (Mm00438023-m1), and *GAPDH* (Mm99999915-g1) which was used as a housekeeping gene (Thermo Fisher, Fremont, CA, USA). Expression of these genes was normalized to *GAPDH* expression and calculated using the formula 2^−ΔΔCt^ and expressed in % *GAPDH* expression.

### 4.9. Statistical Analysis

All data are presented as the mean ± standard deviation (SD) derived from at least three separate independent experiments. Three or more samples were analyzed with analysis of variance (ANOVA) followed by Tukey’s multiple comparison test. Statistical analysis was completed using the GraphPad Prism 6.0.4 software (GraphPad Software, San Diego, CA, USA).

## Supplementary Materials

Supplementary materials can be found at https://www.mdpi.com/1422-0067/21/11/4085/s1.
