# Supplementary material for: HSP70-Mediated NLRP3 Inflammasome Suppression Underlies Reversal of Acute Kidney Injury Following Extracellular Vesicle and Focused Ultrasound Combination Therapy
Source: Int J Mol Sci. 2020 Jun 8;21(11):4085. doi: 10.3390/ijms21114085 (PMC7312940; doi:10.3390/ijms21114085)
Supplement: Supplementary file 1 [file ijms-21-04085-s001.pdf]

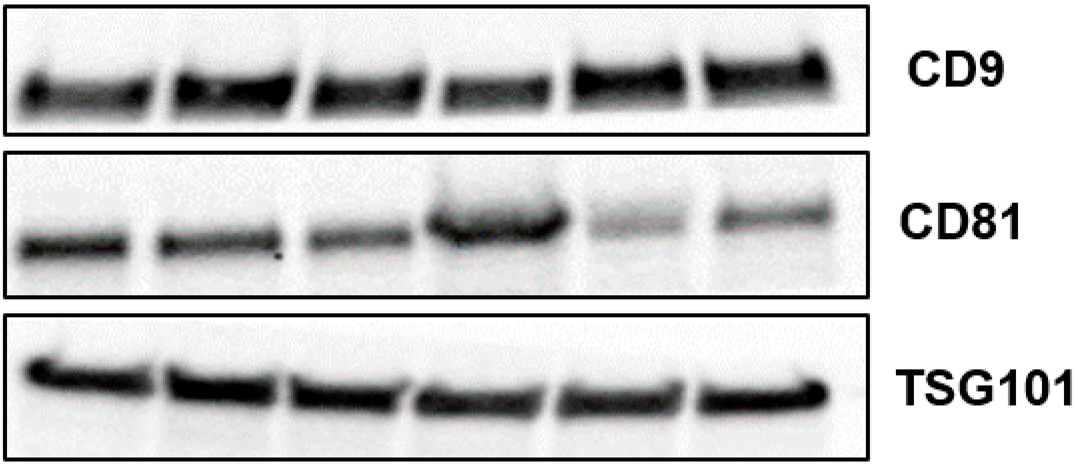

Supplementary Figure S1. Validation of extracellular vesicles. Confirmation of extracellular vesicle surface markers CD9, CD81, and TSG101 by Western blot analysis.
